# Adherence to physical activity recommendations and the influence of socio-demographic correlates – a population-based cross-sectional study

**DOI:** 10.1186/1471-2458-8-367

**Published:** 2008-10-22

**Authors:** Patrick Bergman, Andrej M Grjibovski, Maria Hagströmer, Adrian Bauman, Michael Sjöström

**Affiliations:** 1 Unit for Preventive Nutrition, Department of Biosciences and Nutrition, Karolinska Institute, 141 57 Huddinge, Sweden; 2 Norwegian Institute of Public Health, Pb 4404 Nydalen, 0403 Oslo, Norway; 3 Public Health, School of Public Health, K25 Medical Foundation Building, The University of Sydney, NSW 2006, Australia

## Abstract

**Background:**

Current physical activity guidelines acknowledge the importance of total health enhancing physical activity (HEPA) compared to leisure time physical activity or exercise alone. Assessing total HEPA may result in different levels of adherence to these as well as the strength and/or direction of associations observed between total HEPA and socio-demographic correlates. The aim of this study was to estimate the proportion of the population adhering to the recommendation of at least 30 minutes of HEPA on most days, and to examine the influences of socio-demographic correlates on reaching this recommendation.

**Methods:**

Swedish adults aged 18–74 years (n = 1470) were categorized, based on population data obtained using the IPAQ, into low, moderately and highly physically active categories. Independent associations between the physical activity categories and socio-demographic correlates were studied using a multinomial logistic regression.

**Results:**

Of the subjects, 63% (95% CI: 60.5–65.4) adhered to the HEPA recommendation. Most likely to reach the highly physical active category were those aged < 35 years (OR = 1.8; 95% CI: 1.1–3.3), living in small towns (OR = 1.8; 95% CI: 1.1–2.7) and villages (OR = 2.4; 95% CI: 1.6–3.7), having a BMI between 25.0–29.9 kg/m^2 ^(OR = 2.7; 95% CI: 1.4–5.3) having a BMI < 25 kg/m^2 ^(OR = 2.5; 95% CI: 1.3–4.9), or having very good (OR = 2.1; 95% CI: 1.3–3.3) or excellent self-perceived health (OR = 4.1; 95% CI: 2.4–6.8). Less likely to reach the high category were women (OR = 0.6; 95% CI: 0.5–0.9) and those with a university degree (OR = 0.5; 95% CI: 0.3–0.9). Similar, but less pronounced associations were observed for the moderate group. Gender-specific patterns were also observed.

**Conclusion:**

Almost two-thirds of the Swedish adult population adhered to the physical activity recommendation. Due to a large diversity in levels of physical activity among population subgroups, social-ecological approaches to physical activity promotion may be warranted.

## Background

Physical activity is an important determinant of health and is associated with a lower risk of cardiovascular diseases, obesity, diabetes and certain forms of cancers, i.e. the main causes of death in the developed countries [1]. The importance of the relationship between physical activity and factors that promote or hinder it should therefore be appreciated by public health professionals, from general practitioners to policy planners and politicians.

Global, international and national public health strategies [2,3] to encourage an active lifestyle are based on the widely used recommendation of at least 30 minutes of physical activity on at least moderate intensity on most, preferably all, days per week, i.e. the intensity corresponding to Health Enhancing Physical Activity (HEPA) [4-6].

The adherence to this recommendation at population level, and within groups of the population, is to a great extent unknown, partly because there have not been any valid and reliable methods of assessing HEPA available. Historically, only parts of HEPA have been assessed using one or two general, single-domain questions such as: *How often do you do exercise in your leisure time? *These kinds of questions are not informative since they only indicate the proportion that adhere to the recommendation of intentional exercise alone and disregards all physical activity that may take place in the course of work, transport etc. The development and testing of the International Physical Activity Questionnaire (IPAQ) makes it possible to collect reliable and valid information about physical activity at several intensity levels and across several domains (at home, at work, during transportation and during leisure time) [7].

For a good public health planning and to design and implement interventions to promote physical activity it is important for public health professionals, physicians and politicians, among others, to be aware of which groups in society are the least active and who would benefit the most from increasing their levels of physical activity. Therefore it is important to understand the influence of sociodemographic correlates on physical activity.

This study aims to estimate the adherence to the physical activity recommendation of at least 30 minutes of moderate intensity physical activity on most, preferably all, days of the week and to assess the influence of socio-demographic factors, body mass index and self-perceived health on the physical activity levels in the adult population of Sweden.

## Methods

This is a population-based cross-sectional study and is a part of the International Physical Activity Prevalence Study (IPS). The IPS began in 2002 and is a worldwide collaboration between 20 countries. The aim with IPS is to demonstrate the feasibility of using the International Physical Activity Questionnaire (IPAQ) and to obtain internationally-comparable physical activity prevalence estimates in a large scale pilot study. From the Swedish population, 2500 individuals, of both genders, aged 18–74 years, were sampled at random from the national post and address registry. The short self-administered version of IPAQ was the main instrument for studying physical activity pattern. The questionnaire was mailed to the subjects who returned it completed using a pre-paid return envelope. Consistent with the IPS protocol, all data were collected in October-November 2003. This study was approved by the research ethics committee at Huddinge University Hospital (432/03).

### Assessment of physical activity

HEPA was assessed using the short version of the IPAQ. The short version has been shown to have acceptable test-retest reliability (rho = 0.8) and criterion-related validity, compared with accelerometers (rho = 0.3), in a 12-country evaluation study that included Sweden [7]. The IPAQ assesses HEPA by asking each individual how often (the number of days per week) and for how long (the average time in minutes) he/she has been active at vigorous intensity, moderate intensity and walking. Each intensity was assigned an average MET value. Vigorous was assigned 8.0 MET, moderate 4.0 MET and walking 3.3 MET. The data were scored according to the IPAQ scoring protocol, version 2.0 (accessible at ), with one exception. All subjects that, in one or more intensity category had reported days (frequency) but not time (duration) of physical activity, or vice versa, were recoded as having spent zero time in that intensity category. Otherwise, if one intensity category had contained missing values, it would not have been able to sum up the physical activity and the entire case would have been excluded from analysis due to missing values.

To reduce the effect of known measurement errors of self reports [8-10], and to minimize the effect of the skew in the data, the physical activity was categorised using the IPAQ scoring protocol [11,12]. The cut-off limits, seen in Table 1, for the physical activity categories are based on the current guidelines for physical activity, which state that every adult should be active on most, preferably all days of the week, at moderate intensity accumulating 30 minutes of physical activity. In terms of how the IPAQ measures activity this would be equal to 600 METminutes·week^-1 ^(5 days·30 minutes·4.0 MET), which is the lowest limit for the moderately active category. The cut-off limit for moderately active category also allows a person to be vigorously active for three days per week for 20 minutes (3 days·20 minutes·8.0 MET = 480 METminutes·week^-1^). As the IPAQ measures physical activity across all domains and the physical activity guidelines are based mainly on studies assessing leisure time physical activity, the cut-off for reaching the moderately active category should be viewed as the absolute minimum of physical activity for some health benefit. The higher category aims to include persons that are either doing intentional physical activity three days per week or more, accumulating 1500 METminutes·week^-1^(ca 60 min·3 days·8 MET), or that are accumulating 3000 METminutes·week^-1^. Subjects in this category are believed to be sufficiently active for health benefits across all domains.

**Table 1:** Physical activity categories and cut-off levels based on the IPAQ scoring protocol .

Physical activity category		Cut-off levels
1	Low	- no activity is reported or - some activity is reported but not enough to meet
		categories 2 or 3
		
2	Moderate	- 3 or more days of vigorous activity for at least 20 min. per day or - 5 or more days of moderate intensity activity or walking for at least 30 min. per day or - 5 or more days of any combination of walking, moderate intensity or vigorous intensity activities achieving a minimum of 600 METmin·week^-1^
		
3	High	- 3 or more days of vigorous activity accumulating at least 1500 METmin·week^-1 ^or - 7 days of any combination of walking, moderate or vigorous intensity activities achieving a minimum of 3000 METmin·week^-1^

### Socio-demographic correlates

The participants' age was divided into three categories; 18–34 years, 35–54 years and 55–74 years. Body Mass Index (BMI) was calculated by dividing body weight by height squared (kg/m^2^). A BMI of less than 25 kg/m^2^was classified as normal weight, between 25 and 30 kg/m^2 ^as overweight and more than 30 kg/m^2 ^as obese [13]. Highest educational level achieved was recoded as university/college, high school, basic school and other education. Employment was categorized as employed, student, retired or unemployed/unknown. By income the sample was divided into four groups; < 100 000, 100 000–200 000, 200 000–300 000 and > 300 000 SEK/year (1000 SEK ≈ € 110). Subjects also reported the size of the residential community in which they lived: a large town (> 100 000 inhabitants), a medium-sized town (30 000–100 000), a small-sized town (1000–30 000) or a village (< 1000). The subjects' marital status was classified from four original categories into either married/co-habiting or single (not living with a partner). The participants were classified as current smokers, former smokers or never-smokers. The subjects rated their overall health as one of the following: excellent, very good, good, satisfactory or poor. Due to small numbers in the lowest two groups (satisfactory and poor), they were collapsed into one.

### Statistical analyses

A one sample t-test and a Z-test were used to compare the mean age and gender structure of the sample, respectively, with corresponding characteristics of the adult population of Sweden.

Bivariate relationships between physical activity categories and BMI, socio-demographic correlates and self-rated health were tested by chi-square tests. Independent effects of each independent variable on the categories of physical activity were assessed by a multinomial logistic regression. Crude and adjusted odds ratios (OR) with 95% confidence intervals (CI) were calculated. Adjustments were performed for all studied variables.

The odds ratios were calculated against the reference categories of males, subjects aged 55–74 years, obese, those with basic education, unemployed, smokers, those living in large towns, married/co-habiting, the highest income group and those having satisfactory or poor self-rated health.

In addition, given that the relative importance of the correlates may differ between men and women, multinomial regression models were analysed separately for each gender. Due to the relatively small sample size, the variables for stratified analyses were selected using a backward stepwise procedure, to avoid a suppressor effect. All statistical analyses were performed using SPSS 13.0 (SPSS Inc., Chicago. IL).

## Results

### Adherence to the physical activity recommendation

Altogether, 1470 adults (59%) responded and provided full information on physical activity and were included in the analyses. At the time of the study (2003) the study population had, according to the official statistics of Sweden, the same mean age (46 ± 15 years) as the Swedish population within the same age span (18–74 years). There was a slight overrepresentation of women in this study (52.9%) compared to Sweden in general (50.2%) (p = 0.034).

A total of 63% (95% CI: 60.5 – 65.4) of the study population were classified as either moderately or highly physically active, i.e. adhered to the physical activity recommendation. Of these 37% and 26% reached the moderately and highly physically active category, respectively (Table 2). Slightly more males (64%) than females (61%) adhered to this recommendation. The highest proportion (77%) was found among the subjects rating their health as excellent. The highest proportion of subjects not adhering to the recommendation was found in the obese group, where 59% were classified in the lowest physically active category. Subjects rating their health as satisfactory or poor had the second highest proportion (52%). Significant variation between physical activity categories were seen by gender, age, BMI, education, employment status, size of residential community, marital status and self-perceived health subgroups, but not by income and smoking habits (Table 2).

**Table 2:** The sample characteristics and distribution of physical activity by the IPAQ physical activity categories

	N	%	Low (%)	Moderate (%)	High (%)	p^b^
Gender						< 0.001
Women	777	52.9	38.5	42.3	19.1	
Men	693	47.1	35.5	31.0	33.5	
Age						< 0.001
18–34	395	26.9	29.8	37.6	32.6	
35–54	566	38.5	36.5	40.4	23.1	
55–74	509	34.6	43.6	32.5	23.9	
BMI						
< 25.0	819	55.7	33.8	39.6	26.6	< 0.001
25.0–29.9	508	34.6	37.5	34.6	27.9	
≥ 30.0	118	8.0	58.9	29.5	11.6	
Education						< 0.001
College/university	443	30.1	38.9	42.1	19.0	
High school	632	43.0	32.0	38.3	29.7	
Other	77	5.2	35.2	35.2	29.6	
Basic school	318	21.6	45.4	26.8	27.8	
Employment status						< 0.001
Employed	880	59.9	34.3	39.4	26.4	
Student	126	8.6	25.4	43.4	31.1	
Retired	245	16.7	49.8	29.3	21.0	
Unemployed/unknown	219	14.9	41.5	31.5	27.0	
Income (SEK per year)						0.473
< 100,000	238	16.2	34.1	36.3	29.6	
100,000–200,000	436	27.7	40.4	35.8	23.8	
200,000–300,000	506	34.4	34.1	38.4	27.5	
> 300,000	226	15.4	36.5	37.9	25.6	
Residential community size						0.010
Village	384	26.1	31.2	36.9	32.0	
Small town	355	24.1	35.1	38.4	26.4	
Medium-size town	291	19.8	38.6	35.0	26.4	
Large town	381	25.9	41.5	38.5	20.1	
Marital status						0.110
Single	420	28.6	33.8	36.6	29.6	
Married/Partner	1046	71.2	38.4	37.1	24.5	
Smoking status						0.349
Never smoked	765	52.0	34.9	38.1	27.0	
Former smoker	398	27.1	40.7	33.6	25.3	
Current smoker	293	19.9	37.1	38.6	24.3	
Self-perceived health						< 0.001
Excellent	272	18.5	23.2	36.9	39.9	
Very good	399	27.1	31.7	40.5	27.8	
Good	478	32.5	40.3	38.6	21.1	
Satisfactory or poor	309	21.0	51.8	28.8	19.4	

Total	1470^a^	100^a^	37.1	36.9	26.0	

^a ^Total numbers may not be equal to 1470 and 100% due to missing data in a few variables.

^b ^p for the differences between groups as calculated by chi-square test.

### Influence of socio-demographic factors

Subjects belonging to the highly physically activity category were, in the crude analyses, more likely to be younger than 55, have a BMI below 30 kg/m^2^, have high school education, employed or a student, while those rating their health as very good or better, had increased odds of being in the high physical activity category (Table 3). After adjustment for all studied socio-demographic correlates, males, the age group 18–34 years, those having a BMI below 30 kg/m^2^, those living in a village or a small town, and those reporting a self-rated health as very good or better, had a higher odds of reaching the high category (Table 3).

**Table 3:** Results of multinomial logistic regression for the categories of physical activity by studied socio-demographic correlates

	Moderate				High			
		
	Crude analysis		Adjusted analysis		Crude analysis		Adjusted analysis	
	OR	95% CI	OR	95% CI	OR	95% CI	OR	95% CI
Gender								
Women	1.26	0.98–1.61	1.33	0.99–1.78	0.53	0.40–0.69	0.62	0.45–0.87
Men	1.00		1.00		1.00		1.00	
Age								
18 – 34	1.70	1.23–2.34	1.13	0.71–1.79	2.00	1.42–2.82	1.77	1.06–2.96
35 – 54	1.49	1.12–1.97	1.11	0.77–1.61	1.15	0.84–1.59	1.07	0.70–1.66
55 – 74	1.00		1.00		1.00		1.00	
BMI								
< 25	2.35	1.50–3.68	1.66	1.01–2.75	4.00	2.15–7.46	2.53	1.29–4.94
25.0 – 29.9	1.84	1.16–2.95	1.66	1.00–2.76	3.78	2.00–7.14	2.72	1.39–5.32
≥ 30	1.00		1.00		1.00		1.00	
Education								
College/university	1.83	1.29–2.60	1.18	0.75–1.83	0.80	0.54–1.17	0.52	0.32–0.86
High school	2.03	1.44–2.84	1.43	0.95–2.14	1.37	1.07–2.13	1.01	0.66–1.55
Other	1.69	0.91–3.15	1.20	0.60–2.40	1.51	1.07–2.13	1.25	0.61–2.55
Basic school	1.00		1.00		1.00		1.00	
Employment status								
Employed	1.53	1.05–2.18	1.41	0.92–2.16	1.18	0.81–1.74	1.15	0.72–1.84
Student	2.25	1.30–3.91	2.47	1.27–4.83	1.88	1.05–3.38	1.98	0.95–4.10
Retired	0.77	0.50–1.21	1.06	0.60–1.86	0.65	0.40–1.05	1.00	0.53–1.87
Unemployed/unknown	1.00		1.00		1.00		1.00	
Income (SEK per year)								
< 100,000	1.03	0.66–1.59	0.85	0.47–1.53	1.24	0.77–1.99	0.92	0.47–1.78
100,000 – 200,000	0.85	0.58–1.25	0.90	0.57–1.44	0.84	0.55–1.28	0.93	0.55–1.59
200,000 – 300,000	1.09	0.75–1.57	1.06	0.70–1.59	1.15	0.77–1.74	1.20	0.76–1.93
> 300,000	1.00		1.00		1.00		1.00	
Residential community size								
Village	1.28	0.91–1.79	1.55	1.06–2.28	2.12	1.45–3.10	2.40	1.55–3.72
Small town	1.18	0.84–1.66	1.44	0.99–2.10	1.56	1.05–2.31	1.76	1.13–2.74
Medium-size town	0.98	0.69–1.40	0.98	0.67–1.44	1.42	0.94–2.13	1.44	0.93–2.25
Large town	1.00		1.00		1.00		1.00	
Marital status								
Single	1.12	0.85–1.47	1.08	0.79–1.48	1.37	1.02–1.84	1.21	0.85–1.74
Married/Partner	1.00		1.00		1.00		1.00	
Smoking status								
Never smoked	1.05	0.76–1.45	0.91	0.64–1.30	1.18	0.82–1.70	1.06	0.70–1.59
Former smoker	0.79	0.55–1.14	0.76	0.51–1.13	0.97	0.65–1.44	1.03	0.66–1.62
Current smoker	1.00		1.00		1.00		1.00	
Self-perceived health								
Excellent	2.86	1.88–4.36	2.31	1.44–3.71	4.59	2.94–7.16	4.05	2.42–6.77
Very good	2.30	1.60–3.31	1.81	1.20–2.73	2.34	1.56–3.51	2.07	1.29–3.31
Good	1.72	1.22–2.43	1.49	1.02–2.17	1.40	0.94–2.08	1.24	0.80–1.94
Satisfactory or poor	1.00		1.00		1.00		1.00	

Participants with an education at college/university level were less likely to be in the high category than those with basic education. Women were less likely to be in the high category compared to men both before and after adjustment for other variables.

Subjects in the moderately physically active category were, in crude analyses, likely to be younger than 55, have a BMI below 30 kg/m^2^, have an education level of high school or higher, be employed or a student, or have a self-perceived health of good or better. After adjustment, those with a BMI below 30 kg/m^2^, students, those living in a village or small town and those rating their health as good or better, had increased odds of being in the highly physically active category. Women tended to be more likely to be classified as being in the moderately active category than men, but the results did not reach the level of statistical significance.

### Gender-specific analyses

BMI and marital status were associated with physical activity levels among women, while education and size of residential community were important correlates of physical activity among men. Self-rated health was associated with physical activity in both genders (Table 4).

**Table 4:** Gender specific analyses of physical activity categories

	Moderate		High	
		
	Adjusted OR	95% CI	Adjusted OR	95% CI
WOMEN^a^				
Body Mass Index (kg/m^2^)				
< 25	1.45	0.75–2.80	6.40	1.46–28.10
25.0–29.9	1.65	0.82–3.30	5.95	1.31–26.93
≥ 30	1.00		1.00	
Marital status				
Single	1.32	0.88–1.99	2.00	1.24–3.25
Married/Partner	1.00		1.00	
Self-perceived health				
Excellent	2.81	1.50–5.25	3.72	1.82–7.61
Very good	2.18	1.31–3.66	1.51	0.79–2.89
Good	1.53	0.95–2.46	0.91	0.49–1.71
Satisfactory or poor	1.00		1.00	
				
MEN^a^				
Self-perceived health				
Excellent	2.88	1.48–5.62	5.23	2.67–10.26
Very good	2.31	1.25–4.25	3.57	1.91–6.68
Good	1.92	1.09-3.39	1.82	0.99–3.08
Satisfactory or poor	1.00		1.00	
Education				
College/University	1.22	0.69–2.17	0.51	0.27–0.94
High school	1.69	1.01–2.84	1.74	1.05–2.87
Other	1.51	0.51–4.47	1.59	0.55–4.56
Basic school	1.00		1.00	
Residential community size				
Village	1.44	0.82–2.51	2.68	1.51–4.74
Small town	1.51	0.88–2.60	1.58	0.88–2.85
Medium-size town	0.73	0.40–1.32	1.26	0.69–2.31
Large town	1.00		1.00	

^a^Only variables significantly associated with the outcome are presented. Variables are listed by as selected as a result of the backward elimination procedure in multinominal logistic regression analysis.

## Discussion

Given that the sample is reasonable representative, the acceptable validity of the IPAQ and the inclusion of many potential confounding factors, we feel confident that our findings can be generalised to the Swedish adult population with regards to both the level of adherence to physical activity recommendation and the associations between the studied factors and categories of physical activity. The results suggested that 63%, almost two-thirds of the adult population, adhered to the recommendation, which is similar to comparable data on a convenience sample [14]. The proportion reaching the moderately and highly physical activity category varied by gender, age, BMI, education, employment status, size of residential community and self-rated health categories, were observed. The relative importance of these correlates also differed between genders. The set of plausible findings suggest that the IPAQ is a feasible and valid instrument, which gives information useful for the formulation of a national strategy for the identification of key target groups most needing physical activity promotion strategies.

The use of self reported data with the potential for information bias in relation to physical activity [15] is one of the limitations of our study. Furthermore, the cross-sectional design does not allow making inferences about causality. The response rate of 59%, although relatively low, is close to the response rate in another Swedish study of similar design [16]. The slight overrepresentation of women in this study (53%) compared to Sweden in general (50%) [17], might result in marginally lower estimates of the physical activity in the full sample. The setting of missing values to zero during the data cleaning procedure of the IPAQ might further increase the underestimation of overall physical activity, although this may have just counterbalanced the fact that the IPAQ tends to overestimate physical activity [15].

### Adherence to the physical activity recommendations

The IPAQ has been used for the assessment of physical activity in other large-scale population studies, such as the Eurobarometer 2002 [18] and the WHO 51 country survey of physical inactivity [19]. However as Sweden was not a part of the latter study no comparisons can be made with their results. The Eurobarometer study used face-to-face interviews for collecting data and also a higher cut-off for sufficiently active. However, the cut-off for the lowest physical activity category was identical in both studies which makes it possible to calculate how many of the Eurobarometer participants that adhered to physical activity recommendation using the same cut-off as in our study. In their study, 34% of their subjects were classified in the lowest physical activity category which suggests around 66% adhered to current physical activity recommendation, close to our estimate of 63%.

### Socio-demographic correlates

Associations, not reported previously, between certain socio-demographic variables and total physical activity were found in this study. For example, people of high socio-economic status (high income and/or high education level) have frequently been found to report more leisure time physical activity and exercise than those of low socio-economic status [20-23]. When total HEPA is assessed, having a high income was not associated with categories of physical activity at all, and having a university or college degree was negatively associated with the high physical activity category. While subjects with a higher educational level might do more leisure time exercise, they may have less physically demanding occupations with the result that their overall physical activity is lower than for those with lower educations who may perform more physically demanding work.

Another example was that living in a village or small town was positively associated with physical activity compared with living in a large town (> 100 000 people), especially among the men. This is in contrast to what was found in the USA and Australia [24,25]. However, the studies from the USA and Australia mostly report on leisure time physical activity or walking alone which may explain the observed differences. Furthermore, the USA and Australian data may not easily be compared with Swedish or European data as the physical and cultural environments are different. European studies, on the other hand, show that women living in rural areas of France have higher physical activity levels than their urban counterparts [26]. In Belgium, women living in the outskirts of cities have been shown to be more likely to walk for recreation compared to those living in the inner city [27]. None of these studies found any association for men, while in our study this association was more important for men than for women.

Increasing age has been shown to be negatively associated with physical activity [20-22,28,29]. Sweden has an ageing population and Statistics Sweden estimates that the oldest age group will double by the year of 2050 [17]. The oldest age group is an important group to target with physical activity interventions since they can benefit the most from increased physical activity [30-32].

An inverse association between physical activity and BMI was seen. Those with a BMI over 30, especially among women, had the lowest proportion of reaching the cut-off limit for physical activity to meet the recommendation. Even if it is appealing to draw the conclusion that obesity, at least partly, is explained by low physical activity, there is no convincing evidence that this is the case. The criterion of causality, such as a dose-response relationship between exposure and outcome is missing. Moreover, in the interpretation of the association reverse causality can not be ruled out. Wareham et al reviewed the evidence for the role of physical activity in the prevention of obesity and claimed that only weak associations between low levels of physical activity and weight-gain existed. They also stated that clinical interventions with increased levels of physical activity had small effects on obesity [33]. This area needs further studies to see what role HEPA might play in the prevention of obesity from a public health perspective.

Being married or living with a partner has been shown to be negatively associated with physical activity [34]. Our study supports, in part, these findings, as single women were twice as likely to be in the high category compared to women who were married or co-habited. Unfortunately, no information on parity was included in the questionnaire and therefore we cannot conclude whether marital status *per se *or perhaps having children, hampers physical activity.

Rating self-perceived health highly was an important correlate of physical activity for both men and women as well as in the total sample, supporting previous findings [23,35,36]. Since this is a cross sectional study it is impossible to determine if physical activity leads to increased self-perceived health or if those with high self-perceived health do more physical activity.

Taken together, our findings indicate that levels of physical activity varies substantially between groups in the society. Therefore no particular group can be identified as potential targets for physical activity interventions. A broad approach is needed. To date, many interventions to promote physical activity have shown disappointing results, particularly with regard to the long-term maintenance [37]. More recent public health strategies to promote physical activity is based in social-ecological models [38]. They acknowledge the role of factors external to the individual such as the physical environment, policy factors and social norms [39]. Interventions based in social ecological models is expected to have relatively permanent effects and to affect entire communities or populations [40]. Thus, such models may be the most realistic option to base physical activity interventions on when the variation in physical activity is large.

## Conclusion

The present study is among the first to determine the adherence to the total HEPA recommendation of at least 30 minutes of moderate intensity physical activity on most, preferably all, days of the week, in a population as well as the influence of socio-demographic factors on levels of physical activity. Sixty-three per cent of the adult Swedish population adhered to this recommendation. Given that the levels of physical activity varied greatly between sub-groups of the population, social-ecological approaches to promote physical activity is warranted.

## List of abbreviations

BMI: Body Mass Index; CI: Confidence Interval; HEPA: Health Enhancing Physical Activity; IPAQ: International Physical Activity Questionnaire; IPS: International Physical Activity Prevalence Study; MET: Metabolic Energy Turnover; OR: Odds Ratio; SEK: Swedish Kronor

## Competing interests

The authors declare that they have no competing interests.

## Authors' contributions

MS: Is a member of the international IPAQ core group, who initiated the International Prevalence Study (IPS), and he also initiated the national Swedish study. AB: Is a member of the international IPAQ core group and responsible for the coordination of the international IPS study. PB: Conducted the IPS fieldwork in Sweden and drafted the first version of the manuscript. AMG: Performed the statistical analyses and drafted the manuscript. AB, MH and MS all drafted the manuscript. All authors read and approved the final manuscript.

## Pre-publication history

The pre-publication history for this paper can be accessed here:


